# The Effect of Elevated Body Mass Index on Ischemic Heart Disease Risk: Causal Estimates from a Mendelian Randomisation Approach

**DOI:** 10.1371/journal.pmed.1001212

**Published:** 2012-05-01

**Authors:** Børge G. Nordestgaard, Tom M. Palmer, Marianne Benn, Jeppe Zacho, Anne Tybjærg-Hansen, George Davey Smith, Nicholas J. Timpson

**Affiliations:** 1 Department of Clinical Biochemistry, Herlev Hospital, Copenhagen University Hospital, Copenhagen, Denmark; 2 The Copenhagen General Population Study, Herlev Hospital, Copenhagen University Hospital, Copenhagen, Denmark; 3 The Copenhagen City Heart Study, Bispebjerg Hospital, Copenhagen University Hospital, Copenhagen, Denmark; 4 Faculty of Health Sciences, University of Copenhagen, Copenhagen, Denmark; 5 MRC Centre for Causal Analyses in Translational Epidemiology, University of Bristol, Bristol, United Kingdom; 6 School of Social and Community Medicine, University of Bristol, Bristol, United Kingdom; 7 Department of Clinical Biochemistry, Rigshospitalet, Copenhagen University Hospital, Copenhagen, Denmark; European Academy, Italy

## Abstract

**Background:**

Adiposity, assessed as elevated body mass index (BMI), is associated with increased risk of ischemic heart disease (IHD); however, whether this is causal is unknown. We tested the hypothesis that positive observational associations between BMI and IHD are causal.

**Methods and Findings:**

In 75,627 individuals taken from two population-based and one case-control study in Copenhagen, we measured BMI, ascertained 11,056 IHD events, and genotyped *FTO*(rs9939609), *MC4R*(rs17782313), and *TMEM18*(rs6548238). Using genotypes as a combined allele score in instrumental variable analyses, the causal odds ratio (OR) between BMI and IHD was estimated and compared with observational estimates. The allele score-BMI and the allele score-IHD associations used to estimate the causal OR were also calculated individually. In observational analyses the OR for IHD was 1.26 (95% CI 1.19–1.34) for every 4 kg/m^2^ increase in BMI. A one-unit allele score increase associated with a 0.28 kg/m^2^ (95 CI% 0.20–0.36) increase in BMI and an OR for IHD of 1.03 (95% CI 1.01–1.05) (corresponding to an average 1.68 kg/m^2^ BMI increase and 18% increase in the odds of IHD for those carrying all six BMI increasing alleles). In instrumental variable analysis using the same allele score the causal IHD OR for a 4 kg/m^2^ increase in BMI was 1.52 (95% CI 1.12–2.05).

**Conclusions:**

For every 4 kg/m^2^ increase in BMI, observational estimates suggested a 26% increase in odds for IHD while causal estimates suggested a 52% increase. These data add evidence to support a causal link between increased BMI and IHD risk, though the mechanism may ultimately be through intermediate factors like hypertension, dyslipidemia, and type 2 diabetes. This work has important policy implications for public health, given the continuous nature of the BMI-IHD association and the modifiable nature of BMI. This analysis demonstrates the value of observational studies and their ability to provide unbiased results through inclusion of genetic data avoiding confounding, reverse causation, and bias.

*Please see later in the article for the Editors' Summary*

## Abstract 2

A Mendelian randomization analysis conducted by Børge G. Nordestgaard and colleagues using data from observational studies supports a causal relationship between body mass index and risk for ischemic heart disease.

## Editors' Summary

**Background:**

Ischemic heart disease (IHD; also known as coronary heart disease) is the leading cause of death among adults in developed countries. In the US alone, IHD kills nearly half a million people every year. With age, fatty deposits (atherosclerotic plaques) build up in the walls of the coronary arteries, the blood vessels that supply the heart with oxygen and nutrients. The resultant reduction in the heart's blood supply causes shortness of breath, angina (chest pains that are usually relieved by rest), and potentially fatal heart attacks (myocardial infarctions). Risk factors for IHD include smoking, high blood pressure (hypertension), abnormal amounts of cholesterol and other fat in the blood (dyslipidemia), type 2 diabetes, and being overweight or obese (having excess body fat). Treatments for IHD include lifestyle changes (for example, losing weight) and medications that lower blood pressure and blood cholesterol levels. The narrowed arteries can also be widened using a device called a stent or surgically bypassed.

**Why Was This Study Done?:**

Prospective observational studies have shown an association between a high body mass index (BMI, a measure of body fat that is calculated by dividing a person's weight in kilograms by their height in meters squared; a BMI greater than 30 kg/m^2^ indicates obesity) and an increased risk of IHD. Observational studies, which ask whether people who are exposed to a suspected risk factor develop a specific disease more often than people who are not exposed to the risk factor, cannot prove, however, that changes in BMI/adiposity cause IHD. Obese individuals may share other characteristics that cause both IHD and obesity (confounding) or, rather than obesity causing IHD, IHD may cause obesity (reverse causation). Here, the researchers use “Mendelian randomization” to examine whether elevations in BMI across the lifecourse have a causal impact on IHD risk. Three common genetic variants—*FTO*(rs9939609), *MC4R*(rs17782313), and *TMEM18*(rs6548238)—which have the largest single genetic variant associations with BMI were used in this study. Given that gene variants are inherited essentially randomly with respect to conventional confounding factors and are not subject reverse causation, use of these as instruments (or proxy measures) for variation in BMI as a risk factor (as opposed to measuring BMI directly) allows researchers to comment on whether obesity is causally involved in IHD.

**What Did the Researchers Do and Find?:**

The researchers analyzed data from two population-based studies in which adults were physically examined and answered a lifestyle questionnaire before being followed to see how many developed IDH. They also analyzed data from a case-control study on IDH (in a case-control study, people with a disease are matched with similar people without the disease and the occurrence of risk factors in the patients and controls is compared). Overall, the researchers measured the BMI of 75,627 white individuals, among whom 11,056 already had IDH or developed it, and determined which of the BMI-increasing genetic variants each participant carried. On the basis of the observational data, every 4 kg/m^2^ increase in BMI increased the odds of IDH by 26% (an odds ratio of 1.26). Using a score derived from the combination of the three genetic variants, the researchers confirmed an association between each BMI increasing allele and both BMI (as expected) and IHD (0.28 kg/m^2^ and an odds ratio for IHD of 1.03, respectively). On average, compared to people carrying no BMI-increasing gene variants, people carrying six BMI-increasing gene variants had a 1.68 kg/m^2^ increase in BMI and an 18% increase in IHD risk. To extend this and to essentially reassess the original, observational, relationship between BMI and IHD risk, an “instrumental variable analysis” was used to examine the causal effect of a lifetime change in BMI on the risk of IDH. In this, it was found that for every 4 kg/m^2^ increase in BMI increased the odds of IDH by 52%.

**What Do These Findings Mean?:**

These findings support a causal link between increased BMI and IDH risk, although it may be that BMI affects IDH through intermediate factors such as hypertension, dyslipidemia, and diabetes. The findings also show that observational studies into the impact of elevated BMI on IHD risk were consistent with this, but also that the inclusion of genetic data increases the value of observational studies by making it possible to avoid issues such as confounding and reverse causation. Finally, these findings and those of recent, observational studies have important implications for public-health policy because they show that the association between BMI (which is modifiable by lifestyle changes) and IHD is continuous. That is, any increase in BMI increases the risk of IHD; there is no threshold below which a BMI increase has no effect on IDH risk. Thus, public-health policies that aim to reduce BMI by even moderate levels could substantially reduce the occurrence of IDH in populations.

**Additional Information:**

Please access these Web sites via the online version of this summary at http://dx.doi.org/10.1371/journal.pmed.1001212.

- The American Heart Association provides information about IHD and tips on keeping the heart healthy, including weight management; it also provides personal stories about IHD
- The UK National Health Service Choices website provides information about IHD, including information on prevention and personal stories about IHD
- Information is available from the British Heart Foundation on heart disease and keeping the heart healthy
- The US National Heart Lung and Blood Institute also provides information on IHD (in English and Spanish)
- MedlinePlus provides links to many other sources of information on IHD (in English and Spanish)
- Wikipedia has a page on Mendelian randomization (note: Wikipedia is a free online encyclopedia that anyone can edit; available in several languages)

## Introduction

Observational examination of the prospective association between body mass index (BMI) and ischemic heart disease (IHD) has been undertaken in a range of populations [1]–[3] and has consistently shown a positive relationship between BMI and the risk of IHD and other vascular endpoints [4]–[9]; however, causality in this relationship has not been convincingly demonstrated.

Observational association of BMI with IHD is impaired by confounding [10], reverse causation [11],[12], and bias [12], making it difficult to infer causality. An alternative approach free of confounding, reverse causation, and bias is that of Mendelian randomisation [13]. Mendelian randomisation uses analyses analogous to those in a randomized trial, but where randomization to risk-factor–related genotypes takes place at conception. In the case of IHD, genotypes with the largest known effects on BMI [14], (*FTO*[rs9939609], *MC4R*[rs17782313], and *TMEM18*[rs6548238]), can be used [15] for the reassessment of BMI as a causal risk factor for disease risk.

Genetic variation at the fat mass and obesity related locus (*FTO*) is thought to have a role in the hypothalamic regulation of appetite and food intake or metabolic rate [16]–[21] and has already been used to interrogate the relationship between BMI and acute coronary syndrome, lipid profile in myocardial infarction patients, and mortality [22]–[24]. Furthermore, variation at the same locus has been associated with atherogenic lipid profile and myocardial infarction risk in a manner that suggests an important role for BMI, but that remains unclear [23]. Although, the mechanism of the association between common variation at *MC4R* and *TMEM18* loci and adiposity is currently unclear, these genotypes may still be used to provide evidence of the role of BMI in IHD risk. Indeed, the use of multiple independent genetic instruments for a single risk factor in the undertaking of Mendelian randomisation experiments is recognised as a favourable approach [25].

The aim here was to test the hypothesis that known positive observational relationships between BMI and IHD are causal in two well-sized general population studies (the Copenhagen General Population Study [CGPS]; *n* = 54,613 [3,780 patients with IHD] and the Copenhagen City Heart Study [CCHS]; *n* = 10,474 [2,006 patients with IHD]) and in a large collection of IHD patients with matched controls (the Copenhagen Ischaemic Heart Disease Study [CIHDS]; *n* = 10,540 [5,270 patients with IHD]). Analyses were designed to use an allele score as an unconfounded marker of BMI, and to take advantage of the use of loci *FTO*(rs9939609), *MC4R*(rs17782313), and *TMEM18*(rs6548238) as multiple independent instruments for BMI in efforts to address the potential complicating issue of biological confounding (or pleiotropy). In these analyses, allele score is used as an instrument for assessing the causal relationship between BMI increase and increased IHD risk, which is then directly compared with the observational BMI-IHD association. To evaluate “raw” data, we also show the different parts of the analyses separately, that is, first the BMI-IHD association, second the allele score-BMI association, and third the allele score-IHD association.

## Methods

### Participants

All participants were white and of Danish descent; this information is available through the national Danish Central Person Registry. No participants appeared in more than one of the three studies. The studies were approved by Danish ethical committees and Herlev Hospital.

#### Copenhagen General Population Study

This general population study was initiated in 2003 with ongoing enrolment [26]–[29]. IHD endpoints have been collected from 1976 to May 2009. Individuals were selected on the basis of the national Danish Civil Registration System to reflect the adult Danish population aged 20–100 y. Data were obtained from a questionnaire, a physical examination, blood samples, and from DNA. At the time of genotyping 59,883 participants had been included; of these, 5,270 were used as controls in the CIHDS (see below), leaving 54,613 for analyses in the CGPS.

#### Copenhagen City Heart Study

This prospective general population study was initiated in 1976–1978 with follow-up examinations in 1981–1983, 1991–1994, and 2001–2003 [26]. Participants were recruited and examined exactly as in the CGPS. DNA was available on 10,474 participants attending the 1991–1994 and/or 2001–2003 examinations.

#### Copenhagen Ischemic Heart Disease Study

This case-control study comprises 5,270 patients from the greater Copenhagen area referred for coronary angiography to Copenhagen University Hospital during the period 1991–2009 and 5,270 unmatched controls without IHD randomly sampled from the CGPS. Beside a diagnosis of IHD as described below, these patients also had stenosis/atherosclerosis on coronary angiography and/or a positive exercise electrocardiography test.

### Ischemic Heart Disease

In all three studies, information on diagnosis of IHD (World Health Organization International Classification of Diseases: ICD8 410–414; ICD10 I20–I25) was collected and verified from existing data from 1976 until May 2009 by reviewing all hospital admissions and diagnoses entered in the national Danish Patient Registry and all causes of death entered in the national Danish Causes of Death Registry. Even though some individuals entered into our studies after 1976, we have complete information on all participants on any hospitalisation or death from IHD from 1976 through 2009 through these registries. IHD was angina pectoris and/or myocardial infarction (ICD8 410; ICD10 I21–I22), based on characteristic chest pain, electrocardiographic changes, and/or elevated cardiac enzymes. Follow-up was 100% complete, that is, no individual was lost to follow-up in any of the studies.

### Genotyping

Genotyping was conducted blind to phenotypic data. In the absence of genomewide data, the ABI PRISM 7900HT Sequence Detection system (Applied Biosystems Inc.) was used to genotype the BMI instrument loci: *FTO*(rs9939609), *MC4R*(rs17782313), and *TMEM18*(rs6548238) using TaqMan assays. Polymorphisms were selected as those with the largest known common effect sizes for association with BMI in European populations [14],[30]. Genotyping was verified by DNA sequencing, quality control genotyping re-runs were performed twice, and 99.96% of all available participants were genotyped. To act as an aggregate instrument for BMI, a simple score of 0–6 was constructed as the sum of the number of higher BMI-associated alleles across the three genotypes in each study. To check results from this simplest form of allelic score, weighted allele score was also calculated. This score was generated by taking the weighted sum of the number of BMI “elevating” alleles at each locus scaled per standard deviation increase in BMI. Independent gene variant/BMI effect estimates used were taken from the largest available GWAS for BMI to date [14].

### Body Mass Index

BMI was calculated as measured weight (kg) divided by measured height squared (m^2^). To exclude influence of age and sex on our results, BMI was standardised into age- and sex-adjusted Z-scores within each study separately (Table S1). One Z-score corresponds to a BMI standard deviation of 4 kg/m^2^; thus, for easy interpretation of data, results in Z-scores were converted to BMI values in kg/m^2^. Participants with BMI greater than 50 kg/m^2^ were excluded from observational and instrumental variable estimates. BMI measurements were not available for the patients in the CIHDS, hence observational estimates of the IHD-BMI association are not given for this study.

### Other Covariates

Smoking was categorized from self-reported data as ever versus never smoked, alcohol consumption was categorized as <14/21 or ≥14/21 units per week for women/men (1 unit = 12 g), education as schooling for <10, ≥10 to <13, and ≥13 y, and annual income as <100,000DKK, 100,000DKK–400,000DKK, 400,000DKK–600,000DKK, and >600,000DKK. Use of statins was recorded at examination. Measured systolic and diastolic blood pressure was recorded as described previously [31] and adjusted for antihypertensive medication by adding a constant value of 10 mmHg and systolic blood pressure and 5 mmHg for diastolic blood pressure [32]. Plasma levels of triglycerides, glucose, high-density lipoprotein cholesterol, and low-density lipoprotein cholesterol were measured using standard hospital assays. Time between IHD event and BMI measurement (“event time”) was assessed as a confounding factor in supplementary analyses.

### Statistical Analysis

All analyses were performed in Stata version 11 (StataCorp). For genotypes a deviation from Hardy-Weinberg equilibrium was investigated using a Pearson chi-squared test. Observational estimates of odds ratios (ORs) of IHD per unit increase in standardised BMI, allele score, and potential confounders were estimated using logistic regression. Estimates of the association of standardised BMI with allele score and potential confounders were performed using linear regression. Associations of potential confounders by genotype and allele score were estimated using logistic regression, linear regression, and Pearson's chi-squared test.

Instrumental variable estimates of causal ORs were derived using the Wald-type estimator [33], which involves taking the ratio of the IHD-allele score log OR to the standardised BMI-allele score coefficient and then exponentiating to express as an OR. The standardised BMI-allele score coefficient comes from all individuals from the general population with BMI available, that is, all participants other than those in the CIHDS. Standard errors of Wald-type instrumental variable log ORs were derived using the delta method [34]. Instrumental variable estimates derived from this approach (which is the most efficient use of data) were verified by comparing them to analyses in the CGPS and CCHS using a logistic structural mean model fitted using the three genotypes as separate, weighted, instruments [35]; these models were fitted in the CGPS and CCHS as they require observations of genotypes, BMI, and IHD status for each individual. In these models the joint validity of the multiple instruments was investigated using Hansen's over-identification test [36]. If such a test is rejected it indicates one or more of the instruments may not be valid instruments. As a comparison to using a non-weighted allele score, analyses were also undertaken using a weighted allele score.

In a case-control study like the CIHDS, the allele score-IHD association is valid as in cohort studies like the CGPS and the CCHS because it is not affected by IHD status and represents lifecourse BMI-associated IHD risk. BMI may be affected by IHD, because some patients loose weight after an IHD diagnosis. Therefore, the allele score-BMI association entering into the instrumental variable analyses is best derived from people in the general population as done in the present study. The advantage of including the CIHDS together with the CGPS and the CCHS is that it adds considerable statistical power to the combined analyses, as done by ourselves in previous studies [27]–[29]. Importantly, however, when the CGPS and the CCHS are combined and CIHDS is excluded, the results are similar.

Meta-analysis pooled estimates were obtained using the random effects meta-analysis model implemented in the user-written Stata command “metan” [37]. In each meta-analysis, between-study heterogeneity was assessed using the *I* ^2^ statistic and the Cochran heterogeneity Q test. Meta-analysis of ORs was performed on the log scale. Analysing the three studies as a single dataset was undertaken, but results are not reported as they were similar to those of the meta-analyses throughout.

## Results

The numbers of IHD patients were 3,780 of 54,613 participants in the CGPS (79% prevalent and 21% incident), 2,006 of 10,474 participants in the CCHS (22% prevalent and 78% incident), and 5,270 of 10,540 participants in the CIHDS (100% prevalent). Baseline characteristics of participants in the three studies are shown in Table 1. Genotypes scored for *FTO*(rs9939609), *MC4R*(rs17782313), and *TMEM18*(rs6548238) were in Hardy-Weinberg equilibrium in the three studies and the distribution of the allele score was approximately normal (Table 2).

**Table 1:** Characteristics of the participants in the CGPS, CCHS, and CIHDS by BMI tertile.

Characteristics	CGPS				CCHS				CIHDS				
	Tertile 1	Tertile 2	Tertile 3	Overall	Tertile 1	Tertile 2	Tertile 3	Overall	Tertile 1 Controls	Tertile 2 Controls	Tertile 3 Controls	Controls	Overall
*n*	18,063	18,060	18,060	54,613	3,488	3,488	3,488	10,474	1,758	1,756	1,756	5,270	10,540
BMI (kg/m^2^)	22.3 (21.1–23.2)	25.6 (24.8–26.4)	29.9 (28.5–32.3)	25.6 (23.2–28.5)	21.0 (20.0–21.8)	23.9 (23.3–24.7)	27.9 (26.7–30.0)	23.9 (21.8–26.7)	22.2 (21.1–23.2)	25.4 (24.6–26.2)	29.5 (28.1–31.8)	25.4 (23.2–28.1)	25.5 (23.3–28.1)
Waist hip ratio	0.81 (0.77–0.87)	0.88 (0.82–0.93)	0.93 (0.86–0.98)	0.87 (0.81–0.93)	0.81 (0.76–0.87)	0.87 (0.81–0.93)	0.93 (0.86–0.99)	0.87 (0.80–0.94)	0.81 (0.77–0.87)	0.87 (0.82–0.92)	0.92 (0.86–0.97)	0.87 (0.80–0.93)	0.87 (0.80–0.93)
Women (%)	70.3	48.4	47.7	55.6	70.6	51.8	44.3	55.6	72.8	49.8	47.3	56.6	43.0
Age (y)	54 (44–65)	58 (48–67)	60 (50–68)	57 (47–67)	54 (40–67)	60 (48–70)	64 (54–72)	60 (47–70)	54 (45–64)	56 (46–66)	59 (49–69)	56 (46–66)	60 (51–69)
Ever smoked (%)	56.4	60.7	61.3	59.3	58.0	56.0	53.2	55.7	56.5	59.7	60.5	58.9	59.5
Drinking (%)	73.4	75.2	68.5	72.1	50.9	56.0	53.3	53.4	74.6	75.5	69.5	73.2	59.4
Education (%)^a^	20.3	27.2	37.6	28.4	38.2	50.8	66.0	51.6	20.1	27.5	36.1	27.9	27.9
Education (%)^b^	58.0	55.5	48.9	54.1	46.1	36.8	27.9	36.9	58.3	55.7	50.1	54.7	54.7
Education (%)^c^	21.8	17.3	13.5	17.5	15.8	12.4	6.1	11.4	21.6	16.8	13.8	17.4	17.4
Income (%)^d^	2.0	1.6	2.1	1.9	19.9	16.4	18.1	18.1	2.7	1.4	1.9	2.0	2.0
Income (%)^e^	35.4	36.2	43.9	38.6	52.5	53.9	56.4	54.3	35.8	35.9	41.6	37.7	37.7
Income (%)^f^	38.8	40.7	38.9	39.4	24.3	26.0	22.9	24.4	37.8	41.9	41.0	40.3	40.3
Income (%)^g^	23.8	21.5	15.1	20.1	3.3	3.7	2.7	3.2	23.7	20.8	15.5	20.0	20.0
IHD (%)	4.3	6.9	9.6	6.9	12.4	17.8	27.3	19.2	NA	NA	NA	NA	50.0
Event time (y)	2.1 (1.2–3.6)	2.3 (1.1–3.7)	2.4 (1.0–3.7)	2.3 (1.1–3.7)	15.6 (11.8–16.5)	15.3 (9.3–16.2)	13.5 (6.5–15.9)	15.2 (8.7–16.2)	2.5 (1.5–3.9)	2.6 (1.3–3.9)	2.6 (1.2–3.9)	2.6 (1.3–3.9)	NA
SBP (mmHg)	132 (120–148)	140 (126–154)	145 (132–160)	140 (125–155)	120 (111–132)	127 (117–139)	134 (123–147)	127 (116–140)	130 (120–145)	140 (125–155)	145 (132–160)	140 (125–154)	140 (125–154)
DBP (mmHg)	80 (72–86)	82 (75–90)	86 (80–95)	82 (75–90)	75 (69–83)	80 (73–87)	85 (78–93)	80 (72–88)	80 (72–86)	83 (76–90)	87 (80–95)	83 (75–90)	81 (75–90)
Hypertension (%)	56.5	70.2	83.0	70.0	19.2	32.1	51.1	34.2	54.4	67.8	81.7	68.0	52.7
AH medicine (%)	11.3	17.8	28.3	19.1	1.8	2.4	6.0	3.4	10.9	13.8	24.5	16.4	23.6
Adjusted SBP (mmHg)	132 (120–150)	140 (128–156)	149 (135–164)	140 (126–157)	120 (111–132)	128 (117–140)	135 (123–148)	127 (116–140)	131 (120–148)	140 (125–156)	147 (134–162)	140 (125–156)	140 (126–158)
Adjusted DBP (mmHg)	80 (72–87)	84 (76–90)	88 (80–95)	84 (76–91)	75 (69–83)	80 (73–87)	85 (78–93)	80 (73–88)	84 (76–92)	80 (73–87)	84 (76–91)	84 (76–92)	83 (75–91)
Glucose (mmol/l)	5.0 (4.6–5.5)	5.1 (4.7–5.6)	5.2 (4.8–5.8)	5.1 (4.7–5.6)	5.7 (5.1–6.3)	5.9 (5.3–6.5)	6.1 (5.6–6.8)	5.9 (5.3–6.6)	5.0 (4.6–5.5)	5.0 (4.7–5.5)	5.2 (4.8–5.8)	5.1 (4.7–5.6)	5.1 (4.7–5.6)
LDL cholesterol (mmol/l)	3.0 (2.4–3.6)	3.2 (2.6,3.9)	3.4 (2.7–4.0)	3.2 (2.6–3.9)	3.3 (2.6–4.2)	3.6 (2.9–4.4)	3.8 (3.1–4.6)	3.6 (2.9–4.4)	3.0 (2.4–3.6)	3.2 (2.7–3.9)	3.4 (2.8–4.0)	3.2 (2.6–3.9)	3.1 (2.4–3.8)
HDL cholesterol (mmol/l)	1.8 (1.5–2.2)	1.6 (1.3–1.9)	1.4 (1.1–1.7)	1.6 (1.3–1.9)	1.6 (1.3–1.9)	1.5 (1.2–1.7)	1.3 (1.1–1.6)	1.5 (1.2–1.7)	1.8 (1.5–2.2)	1.6 (1.3–1.9)	1.4 (1.1–1.7)	1.6 (1.3–2.0)	1.4 (1.1–1.8)
Triglycerides (mmol/l)	1.1 (0.8–1.5)	1.4 (1.0–2.1)	1.8 (1.3–2.6)	1.4 (1.0–2.1)	1.1 (0.8–1.5)	1.3 (0.9–1.8)	1.7 (1.2–2.5)	1.3 (0.9–1.9)	1.1 (0.8–1.5)	1.4 (1.0–2.1)	1.8 (1.3–2.6)	1.4 (1.0–2.1)	1.5 (1.0–2.2)

Data are from study enrolment in 2003–2009 in the CGPS, from the 1991–1994 or 2001–2003 examinations of the CCHS when DNA was collected, and from study enrolment in 1991–2009 in the CIDHS. Values are median and interquartile range or number of participants and percentages. Drinking alcohol represented by <14/21; ≥14/21 units per week for women/men at the time of examination. Event time is the absolute value of the difference between age at IHD event and age at measurement of BMI (y). SBP and DBP adjusted by adding 10 and 5 mmHg to each, respectively, if patient on AH medication. To convert glucose values in mmol/l to mg/dl, multiply by 18. To convert LDL and HDL cholesterol values in mmol/l to mg/dl, multiply by 38.6. To convert triglyceride values in mmol/l to mg/dl, multiply by 88. In CIHDS, education and income are only available in controls.

a <10 y of schooling.

b ≥10 to <13 y of schooling.

c ≥13 y of schooling.

d <100,000DKK annual income.

e 100,000DKK–400,000DKK annual income.

f 400,000–600,000DKK annual income.

g >600,000DKK annual income.

AH, antihypertensive; DBP, diastolic blood pressure; HDL, high density lipoprotein; LDL, low density lipoprotein; SBP, systolic blood pressure.

**Table 2:** Distribution of *FTO*, *MC4R*, and *TMEM18* genotypes in the CGPS, CCHS, and CIHDS.

Genotype or Allele Score	0	1	2	3	4	5	6
**Genotype**							
**CPGS**							
*FTO*(rs9939609), *n* (%)	17,846 (35.2)	24,595 (48.5)	8,307 (16.4)				
*MC4R*(rs17782313), *n* (%)	28,797 (56.7)	18,862 (37.2)	3,092 (6.1)				
*TMEM18*(rs6548238), *n* (%)	1,517 (2.9)	15,036 (28.4)	36,435 (68.9)				
**CCHS**							
*FTO*(rs9939609), *n* (%)	3,744 (35.8)	4,981 (47.6)	1,748 (16.7)				
*MC4R*(rs17782313), *n* (%)	5,998 (57.3)	3,841 (36.7)	635 (6.1)				
*TMEM18*(rs6548238), *n* (%)	316 (3.0)	2,975 (28.4)	7,180 (68.6)				
**CIHDS**							
*FTO*(rs9939609), *n* (%)	3,613 (34.3)	5,159 (49.0)	1,768 (16.8)				
*MC4R*(rs17782313), *n* (%)	5,913 (56.1)	3,930 (37.3)	697 (6.6)				
*TMEM18*(rs6548238), *n* (%)	279 (2.7)	2,955 (28.0)	7,306 (69.3)				
**Allele score**							
**CPGS**, *n* (%)	58 (0.6)	738 (7.1)	2,782 (26.6)	3,768 (36.0)	2,386 (22.8)	658 (6.3)	80 (0.8)
**CCHS**, *n* (%)	294 (0.6)	3,452 (6.8)	13,203 (26.0)	18,578 (36.6)	11,556 (22.8)	3,283 (6.5)	349 (0.7)
**CIHDS**, *n* (%)	55 (0.5)	682 (6.5)	2,672 (25.4)	3,831 (36.4)	2,506 (23.8)	721 (6.8)	73 (0.7)

All SNPs adhere to Hardy-Weinberg Equilibrium (*p*>0.2). *FTO(rs9939609)* genotypes, 0 (TT), 1 (AT), 2 (AA); *MC4R(rs17782313)* genotypes 0 (TT), 1 (CT), 2 (CC); *TMEM18(rs6548238)* genotypes 0 (TT), 1 (CT), 2 (CC). Allele score constructed from the following genotypes: *FTO*(rs9939609), 0 (TT), 1 (AT), 2 (AA); *MC4R*(rs17782313), 0 (TT), 1 (CT), 2 (CC); *TMEM18*(rs6548238), 0 (TT), 1 (CT), 2 (CC).

### BMI and IHD Risk: Observational Estimate

**Figure 1:**
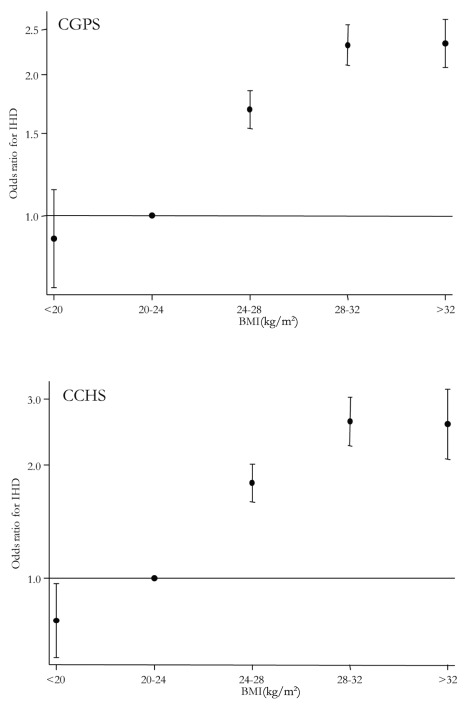
ORs and 95% CIs for IHD by BMI categories in the CGPS and the CCHS.

**Figure 2:**
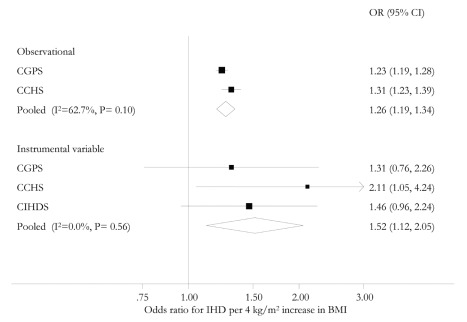
Meta-analysis forest plots of observational and instrumental variable causal estimates using allele score of the relationship between IHD and BMI. The ORs are for a 4 kg/m^2^ increase in BMI.

In the general population, for one standard deviation (4 kg/m^2^) increase in BMI, the observational ORs for IHD were 1.23 (95% CI 1.19–1.28) for the CGPS and 1.31 (95% CI 1.23–1.39) for the CCHS (Figure 1). The meta-analysis pooled OR was 1.26 (95% CI 1.19–1.34), and there was some evidence of between-study heterogeneity (*I* ^2^ = 63; *p* = 0.10), but this was incorporated in the random effects model (Figure 2). These observational associations were unaffected on adjustment for the potential confounders of sex, smoking status, drinking status, years of education, and income level and attenuated on additional adjustment for event time (Table 3). If ever smokers were separated into current and former smokers and drinkers into moderate and heavy drinkers, observational associations were unaffected. Where available (CPGS), analysis including adjustment for statin use showed evidence for an attenuation in estimates of the relationship between BMI and IHD risk (adjusted OR for IHD per 4 kg/m^2^ increase in BMI 1.12 [1.08–1.16]).

**Table 3:** Observational ORs for IHD per 4 kg/m^2^ increase in BMI.

Study	OR (95% CI)	OR (95% CI)^a^	OR (95% CI)^b^
CGPS	1.23 (1.19–1.28)	1.21 (1.17–1.24)	1.22 (1.18–1.26)
CCHS	1.31 (1.23–1.39)	1.31 (1.24–1.38)	1.22 (1.15–1.29)
Pooled	1.27 (1.19–1.34)	1.25 (1.16–1.36)	1.22 (1.18–1.26)

Pooled estimates from fixed effects meta-analysis.

a Adjusted for sex, smoking status, drinking status, years of education, income level.

b Adjusted for sex, smoking status, drinking status, years of education, income level, and event time.

### Genotype and Allele Score Associations with BMI

**Figure 3:**
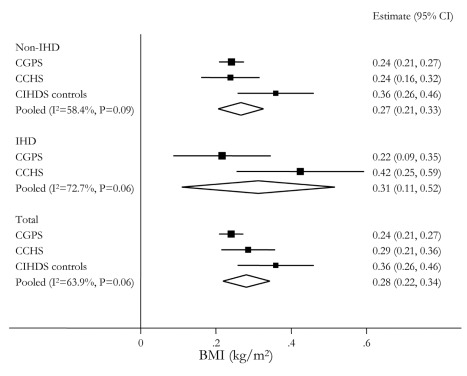
Meta-analysis forest plots of the relationships between *FTO* rs9939609, *MC4R* rs17782313, and *TMEM18* rs6548238 allele score and BMI. Analyses are stratified by IHD status.

Pooled across the three studies, each additional adiposity-related allele from the allele score was associated with a 0.28 kg/m^2^ (95% CI 0.22–0.34) increase in BMI (Figure 3), corresponding to a 1.68 kg/m^2^ (95% CI 1.31–2.06) BMI increase for a comparison of the minimum (0) to maximum (6) carriage of adiposity increasing alleles. These results were similar across cases and controls in the three separate studies.

### Associations with Confounding Factors

In an analysis such as this one, it is important to examine whether potential confounding factors could be part of the explanation behind an observational BMI-IHD association, or behind a causal BMI-IHD association estimated using allele score in instrumental variable analysis. For a potential confounder to be part of the explanation behind an association, the factor in question needs to associate both with the exposure and the outcome.

We therefore examined both the association between potentially confounding factors (sex, age, smoking status, drinking status, education, income, and event time) and our primary exposure (BMI), our primary outcome (IHD), and our instrumental variables (genotypes and allele score). For both our primary outcome and exposure, there was strong evidence for association between both BMI, IHD, and drinking, education, income, and event time (Tables S2 and S3). In contrast, genotypes at *FTO*(rs9939609), *MC4R*(rs17782313), and *TMEM18*(rs6548238) and the allele score demonstrated no reliable evidence of association with potential confounders (Table 4).

**Table 4:** Associations of potential confounders with genotypes in the three studies.

Potential Confounders with Genotypes or Allele Score	OR (95% CI) on Logistic Regression^a^ or on Linear Regression^b^		
	CGPS	CCHS	CIHDS
**Sex** a			
*FTO* (rs9939609)	1.02 (0.99–1.04)	0.95 (0.90–1.00)	1.01 (0.95–1.06)
*MC4R* (rs17782313)	0.99 (0.96–1.01)	0.95 (0.89–1.01)	1.06 (1.00–1.13)
*TMEM18* (rs6548238)	0.99 (0.96–1.03)	0.95 (0.89–1.03)	1.02 (0.94–1.09)
Allele score	1.00 (0.98–1.02)	0.95 (0.92–0.99)	1.03 (0.99–1.07)
**Ever smoked** a			
*FTO* (rs9939609)	1.02 (1.00–1.05)	0.95 (0.90–1.01)	1.03 (0.97–1.09)
*MC4R* (rs17782313)	1.01 (0.98–1.04)	1.02 (0.95–1.08)	0.98 (0.91–1.04)
*TMEM18* (rs6548238)	1.03 (1.00–1.06)	1.05 (0.97–1.12)	1.00 (0.92–1.08)
Allele score	1.02 (1.00–1.04)	1.00 (0.96–1.03)	1.00 (0.96–1.04)
**Drinking** b			
*FTO* (rs9939609)	0.97 (0.95–1.00)	0.93 (0.88–0.99)	1.00 (0.94–1.06)
*MC4R* (rs17782313)	0.99 (0.96–1.03)	0.95 (0.89–1.01)	0.99 (0.92–1.06)
*TMEM18* (rs6548238)	0.98 (0.95–1.02)	1.03 (0.96–1.11)	1.01 (0.93–1.10)
Allele score	0.98 (0.97–1.00)	0.96 (0.93–1.00)	1.00 (0.96–1.04)
**Age (y)** b			
*FT*O (rs9939609)	−0.02 (−0.19 to 0.15)	0.06 (−0.38 to 0.50)	0.09 (−0.26 to 0.44)
*MC4R* (rs17782313)	0.10 (−0.10 to 0.30)	0.15 (−0.36 to 0.65)	0.25 (−0.14 to 0.63)
*TMEM18* (rs6548238)	−0.05 (−0.27 to 0.16)	0.69 (0.11–1.27)	0.04 (−0.41 to 0.50)
Allele score	0.02 (−0.10 to 0.13)	0.24 (−0.05 to 0.53)	0.13 (−0.09 to 0.36)
**Event time** b			
*FTO* (rs9939609)	0.00 (−0.03 to 0.03)	0.01 (−0.14 to 0.15)	0.02 (−0.04 to 0.08)
*MC4R* (rs17782313)	−0.01 (−0.04 to 0.02)	0.05 (−0.12 to 0.21)	−0.03 (−0.09 to 0.04)
*TMEM18* (rs6548238)	0.02 (−0.02 to 0.05)	−0.16 (−0.35 to 0.03)	0.05 (−0.03 to 0.12)
Allele score	0.00 (−0.02 to 0.02)	−0.02 (−0.12 to 0.08)	0.01 (−0.03 to 0.05)
**Education** a			
*FTO* (rs9939609)	0.99 (0.96–1.02)	0.92 (0.85–1.01)	0.95 (0.86–1.04)
*MC4R* (rs17782313)	1.00 (0.97–1.04)	0.93 (0.84–1.03)	0.94 (0.84–1.05)
*TMEM18* (rs6548238)	1.00 (0.96–1.04)	0.95 (0.85–1.06)	0.91 (0.80–1.04)
Allele score	0.99 (0.97–1.02)	0.93 (0.88–0.99)	0.94 (0.88–1.00)
**Income** a			
*FTO* (rs9939609)	0.99 (0.96–1.03)	0.98 (0.85–1.13)	0.99 (0.93–1.06)
*MC4R* (rs17782313)	0.98 (0.95–1.02)	0.96 (0.81–1.13)	1.03 (0.95–1.11)
*TMEM18* (rs6548238)	0.94 (0.91–0.98)	0.90 (0.75–1.08)	1.00 (0.91–1.10)
Allele score	0.98 (0.96–1.00)	0.96 (0.87–1.05)	1.01 (0.96–1.05)

Drinking represented by <14/21; ≥14/21 units per week for women/men at the time of examination. Education represented by less or more than 13 y schooling. Income represented by annual income less or more than 400,000DKK. Event time is absolute difference between age at IHD event and age at measurement of BMI (y).

a **Logistic regression.**

b **Linear regression.**

In aggregate, these data suggest that several factors likely could confound the observational BMI-IHD association. In contrast, it is unlikely that these same factors should confound the instrumental variable analyses assessing the causal BMI-IHD association, as these factors were not associated with genotype or allele score.

### Allele Score and IHD Risk

**Figure 4:**
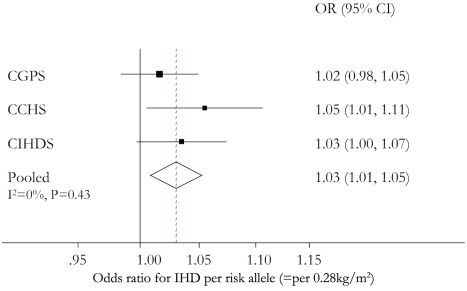
Meta-analysis forest plots of the relationships between *FTO* rs9939609, *MC4R* rs17782313, and *TMEM18* rs6548238 allele score and IHD.

**Figure 5:**
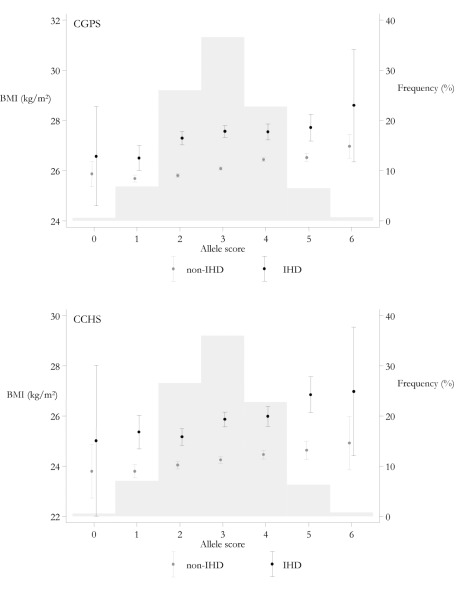
Mean BMI and 95% CIs by allele score and IHD status and distribution of allele score in the CGPS and CCHS. Left *y*-axis and dot with 95% CI depict BMI values as a function of allele score. Right *y*-axis and histogram depict frequency in percent of the different allele scores.

In the CGPS the OR for IHD per risk allele, that is per 0.28 kg/m^2^ increase, was 1.02 (95% CI 0.98–1.05), in the CCHS 1.05 (95% CI 1.01–1.11), and in the CIHDS 1.03 (95% CI 1.00–1.07). The meta-analysed OR per risk allele was 1.03 (95% CI 1.01–1.05), with no evidence of between-study heterogeneity (*I* ^2^ = 0, *p* = 0.43) (Figure 4). This finding corresponds to an OR for IHD of 1.20 (95% CI 1.05–1.36) for a maximum allele score comparison of 0–6 adiposity increasing alleles corresponding to a 1.68 kg/m^2^ increase. The increasing trend in BMI with allele score, distribution of allele score, and the relative differences in BMI by IHD status can be seen simultaneously in Figure 5. The relatively high value of BMI at 0 allele score is likely due to the play of chance, given that there are relatively few individuals with this allele score. The absolute BMI values are higher in the CGPS because participants were recruited approximately 15 y after the CCHS, during which time Danish people on average become heavier for height. Where available (CPGS), analysis including adjustment for statin use showed no strong evidence of attenuation, (adjusted OR for IHD per risk allele 1.01 [95% CI 0.98–1.05]).

### BMI and IHD Risk: Instrumental Variable Causal Estimate Using Allele Score

An instrumental variable analysis using allele score examines the causal effect of a lifecourse change in BMI on the risk of IHD. The instrumental variable estimate of the causal relationship between a 4 kg/m^2^ increase in BMI and IHD showed an OR of 1.31 (95% CI 0.76–2.26) in the CGPS, 2.11 (95% CI 1.05–4.24) in the CCHS, and 1.46 (95% CI 0.96–2.24) in the CIHDS. The meta-analysed causal OR was 1.52 (95% CI 1.12–2.05), with no evidence of between-study heterogeneity (*I* ^2^ = 0, *p* = 0.56) (Figure 2). These results were similar using a weighted allele score (Figure S1), rather than the non-weighted model used above. There was no strong evidence of a difference between instrumental variable and observational ORs (*p* = 0.25 for difference). Where available (CPGS), analysis including adjustment for statin use showed that, in contrast to observational analysis, there was no evidence for an attenuation of the estimate (adjusted OR for IHD per 4 kg/m^2^ increase in BMI 1.28 [0.69–2.37]).

Instrumental variable estimates based on the use of individual SNPs gave broadly similar results, but with reduced statistical power (Figure S2). Also instrumental variable estimates from a logistic structural mean model gave similar estimated causal ORs (Figure S3). In these models there was no evidence against the joint validity of using these SNPs as multiple instruments (CGPS, *p* = 0.97; CCHS, *p* = 0.91).

**Figure 6:**
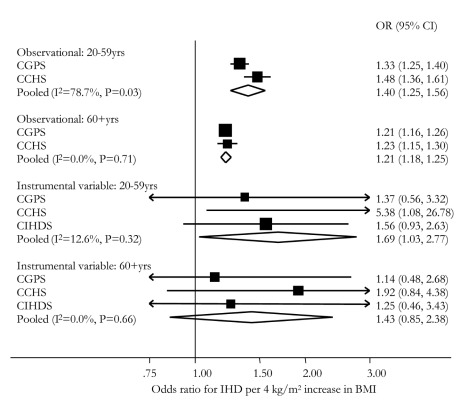
Meta-analysis forest plots of observational and instrumental variable estimates of the relationship between IHD and BMI stratified by age group.

As the relationships between BMI and health outcomes can be attenuated with age, we performed subgroup analyses in individuals above and below age 60 y. There was evidence of smaller observational ORs for IHD in those above versus below 60 y (*p* = 0.02 for difference). This finding was reflected in evidence for interaction between age group and BMI when included in the logistic regression of IHD on BMI and age group (*p* = 0.01 [CGPS], *p*<0.001 [CCHS] for interaction). Instrumental variable estimates gave no evidence of difference by age (Figure 6).

### BMI and IHD Risk: Observational Versus Instrumental Variable Causal Estimate Using Allele Score

In summary, in observational analyses for every 4 kg/m^2^ increase in BMI, the OR for IHD was 1.26 (95% CI 1.19–1.34), corresponding to a 26% increased IHD risk (Figure 2). For comparison, in instrumental variable analysis using the allele score the causal IHD ORs for a 4 kg/m^2^ increase in BMI was 1.52 (95% CI 1.12–2.05), corresponding to a 52% increased IHD risk.

## Discussion

In two large studies of the general population, observational estimates suggested a 26% increase in risk of IHD for every 4 kg/m^2^ increase in BMI. A unit change of an allelic score combining genotypes from three established genetic associates of BMI was associated with a 0.28 kg/m^2^ change in BMI, a change neither correlated with classic confounding features nor affected by reverse causation. Using this as an instrument for lifecourse BMI difference in 75,627 Danish individuals from the same general population studies and a further case-control series, instrumental variable analysis was employed to re-estimate the causal relationship between BMI and IHD risk. In doing this, estimates suggest that the same increase in BMI is causally related to an increased risk of IHD consistent with observational estimates, if not greater. Whilst features such as statin use appear to impact observational estimates, those based on genetic instruments for BMI appear consistent across sub-analyses. Importantly, we not only qualify the likely causal role of BMI (rather than just an observational associate), we are able to quantify this effect. It should be mentioned that the causal relationship between increased BMI and increased risk of IHD may be realised through intermediate factors like hypertension, dyslipidemia, and type 2 diabetes.

The design of this study and the use of genetic variation as a proxy marker for elevated BMI lead to additional discussion points. Firstly, the use of effective instruments for BMI has allowed for forms of study bias to be effectively accounted for. For example, observational relationships between BMI and IHD risk tend to increase with time from BMI measurement largely as a result of tracking and the natural progression of BMI with age [2]. In contrast to this, where IHD events are recorded before BMI, reverse causation can reduce the strength of relationships between BMI and IHD, though illness-induced weight loss adds further complication to the assessment of relationships between body weight and mortality [11]. Genetic instruments are not related to this time difference and the use of these as proxy markers for BMI removes the limitations that can be brought about by the existence of these biases. Use of genetic variation as a proxy measure for lifecourse BMI change also helps to avoid regression dilution bias, or the artefactual underestimation of epidemiological associations owing to a failure of baseline measurements to reliably collect the “usual” levels of an exposure [38]. Existing studies have made efforts to account for this and other “confounding at baseline” issues [2],[3],[6]; however, these effects are difficult to quantify. Owing to the consistent nature of genotype effects through adult life [14],[39], this type of loss in measurement precision is minimised. Indeed, as a result of Mendelian randomisation-derived estimates modelling lifecourse differences in BMI, we would anticipate effect estimates that can be larger than those from more conventional observational approaches, as seen in this case.

Despite these benefits, there are a number of potential limitations in Mendelian randomisation studies like the present one [13],[40],[41]. However, the complicating effect of population stratification in Mendelian randomisation studies is likely to have been avoided through the use of an ethnically homogenous white population in the present study. Also, pleiotropy and the potentially confounding effects of linkage disequilibrium are likely avoided owing to the use of multiple genetic polymorphisms, each associated with increased BMI and each acting on BMI independently and via different pathways. Nevertheless, canalisation cannot be completely excluded as a limitation of the present study; however, as canalisation theoretically acts to buffer the effect of genetic deviations, it can obscure associations between genes and IHD, but it is unlikely to explain away the results in this study. Also, although the analyses provide insight into the likely causal effect of lifecourse elevations in BMI, we are not well placed to comment on the impact of acute changes. Lastly, there are known limitations to the use of BMI as a marker for adiposity or other possible anthropometrically related aetiological contributions to IHD risk, and using alternative measures may prove more informative [42]. Nevertheless, these potential limitations to BMI cannot explain the results in this study.

In the context of available evidence concerning the causal role of BMI as an intermediate risk factor for IHD, we can speculate that the explanation for the causal association is straightforward: increased BMI contributes causally to well-known cardiovascular risk factors including hypertension, dyslipidemia, and type 2 diabetes, factors that may then go on to cause the observed increased risk of IHD. Similar evidence supporting the role of elevated BMI in the generation of a common risk profile is emerging [23],[24],[31],[43],[44] and in combination with the agreement of these findings with those of observational studies, an important role for BMI is evident from work using other techniques to avoid the problems of confounding and reverse causation [22],[45],[46].

In conclusion, for every 4 kg/m^2^ increase in BMI, observational estimates suggested a 26% increase in IHD risk with instrumental variable analysis suggesting a causal 52% increase in IHD risk. These data add novel evidence to support a causal link between increased BMI and increased IHD risk, while the mechanism of this effect is likely to be operating through intermediate factors. In the context of recent, high impact, observational findings, this work has important policy implications for public health given the continuous nature of the BMI-IHD association, the modifiable nature of BMI, and the likely benefits of reducing BMI even by moderate levels. Finally, this analysis demonstrates the value of observational studies and their ability to provide essentially unbiased results because of inclusion of genetic data avoiding confounding, reverse causation, and bias.

## Supporting Information

Figure S1 Meta-analysis forest plots of observational and instrumental variable estimates using a weighted allele score of the relationship between IHD and standardised BMI.
(TIF)

Figure S2 Meta-analysis forest plots of instrumental variable causal estimates of the relationship between IHD and BMI stratified by genotype. *FTO* rs9939609, *MC4R* rs17782313, and *TMEM18* rs6548238.
(TIF)

Figure S3 Meta-analysis of logistic structural mean model causal OR estimates of IHD risk per 4 kg/m^2^ increase in BMI in the CGPS and CCHS. Logistic structural mean models fitted using *FTO,* *MC4R,* and *TMEM18* genotypes as multiple instruments, with each genotype under an additive model. The first stage association model was fitted with an intercept and the main effects of standardised BMI and each of the three genotypes.
(TIF)

Table S1 Means and standard deviations of BMI by 5-y age band and sex used to generate standardised BMI in the CGPS and CCHS.
(DOCX)

Table S2 Associations of potential confounders with standardised BMI in the three studies.
(DOCX)

Table S3 Associations of potential confounders with IHD in the three studies.
(DOCX)

## Abbreviations

BMI: body mass index
CCHS: Copenhagen City Heart Study
CGPS: Copenhagen General Population Study
CIHDS: Copenhagen Ischaemic Heart Disease Study
IHD: ischemic heart disease
