# Supplementary figures and images for: The Effect of Elevated Body Mass Index on Ischemic Heart Disease Risk: Causal Estimates from a Mendelian Randomisation Approach
Source: PLoS Med. 2012 May 1;9(5):e1001212. doi: 10.1371/journal.pmed.1001212 (PMC3341326; doi:10.1371/journal.pmed.1001212)

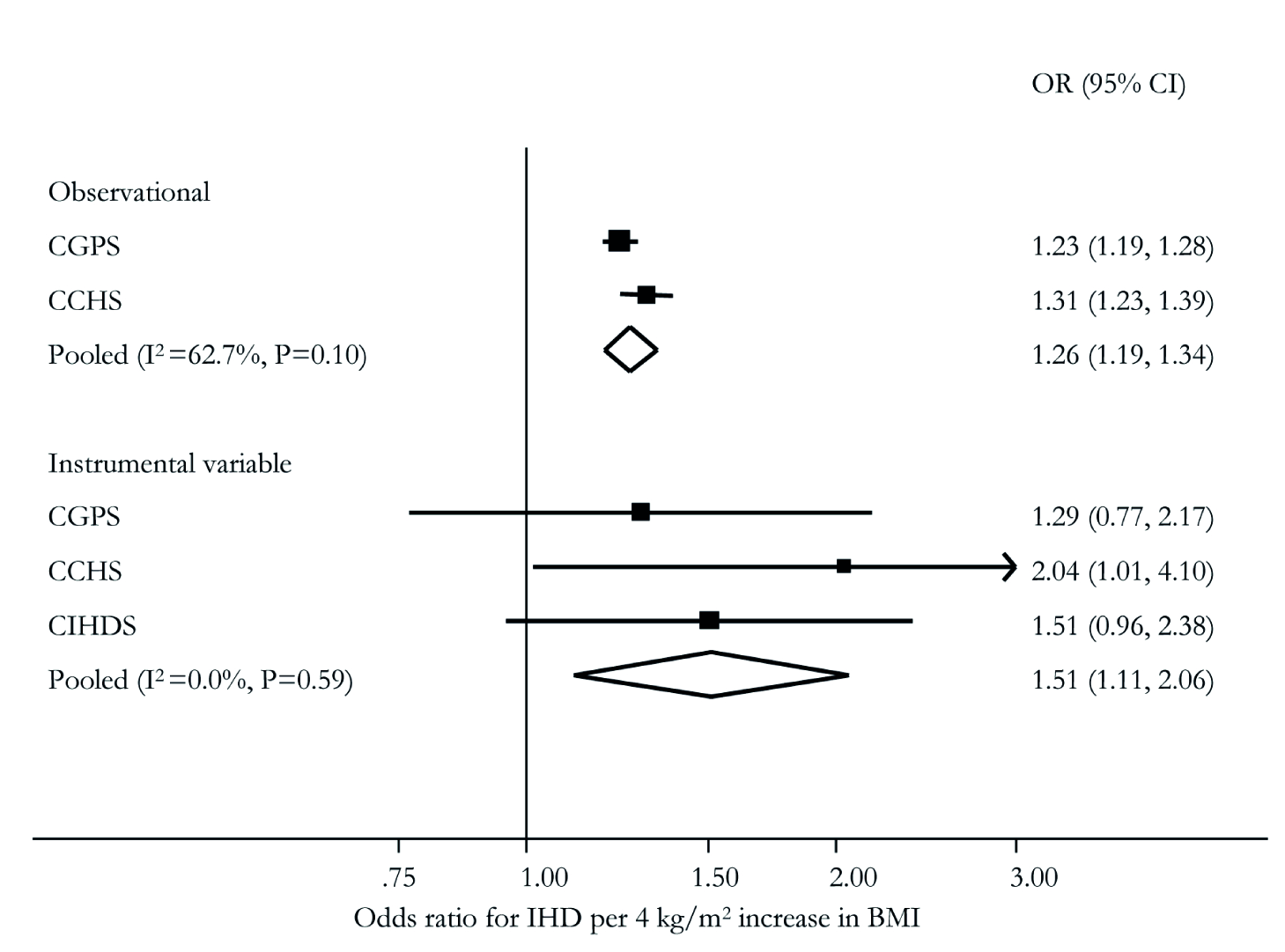

Supplement: Figure S1 — Meta-analysis forest plots of observational and instrumental variable estimates using a weighted allele score of the relationship between IHD and standardised BMI. (TIF) [file pmed.1001212.s001.tif]

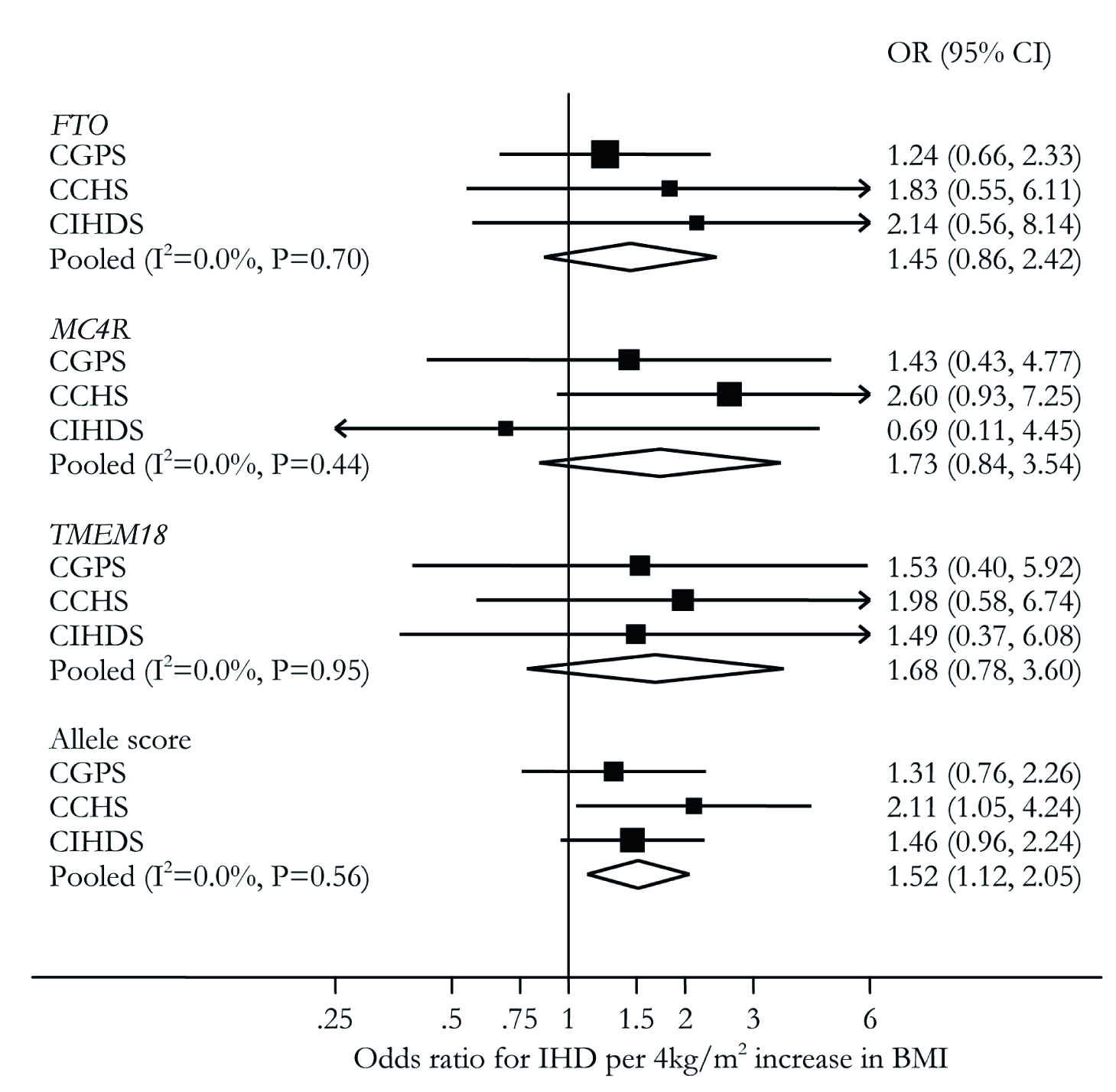

Supplement: Figure S2 — Meta-analysis forest plots of instrumental variable causal estimates of the relationship between IHD and BMI stratified by genotype. FTO rs9939609, MC4R rs17782313, and TMEM18 rs6548238. (TIF) [file pmed.1001212.s002.tif]

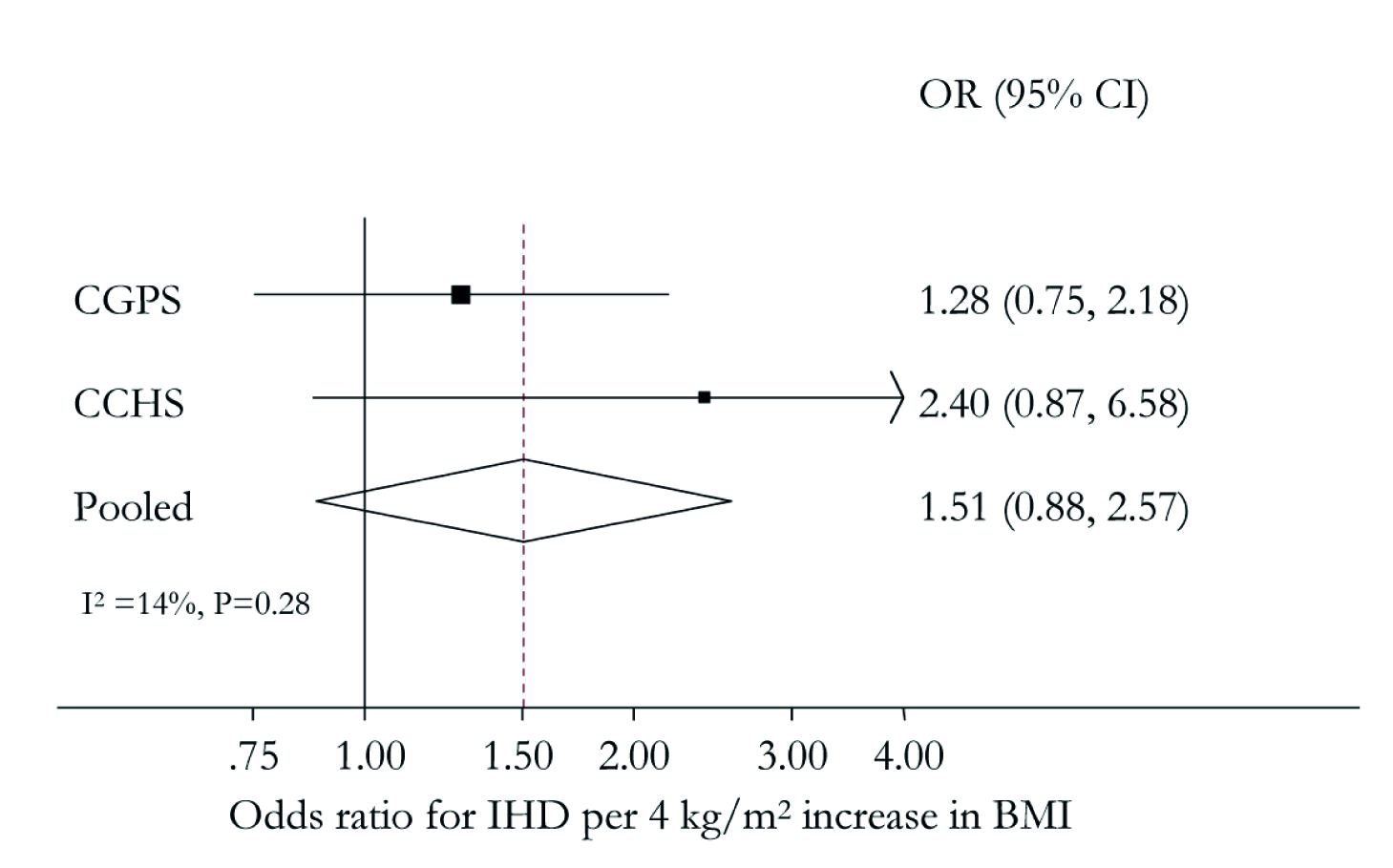

Supplement: Figure S3 — Meta-analysis of logistic structural mean model causal OR estimates of IHD risk per 4 kg/m2 increase in BMI in the CGPS and CCHS. Logistic structural mean models fitted using FTO, MC4R, and TMEM18 genotypes as multiple instruments, with each genotype under an additive model. The first stage association model was fitted with an intercept and the main effects of standardised BMI and each of the three genotypes. (TIF) [file pmed.1001212.s003.tif]
