# Supplementary material for: The Effect of Elevated Body Mass Index on Ischemic Heart Disease Risk: Causal Estimates from a Mendelian Randomisation Approach
Source: PLoS Med. 2012 May 1;9(5):e1001212. doi: 10.1371/journal.pmed.1001212 (PMC3341326; doi:10.1371/journal.pmed.1001212)
Supplement: Table S1 — Means and standard deviations of BMI by 5-y age band and sex used to generate standardised BMI in the CGPS and CCHS. (DOCX) [file pmed.1001212.s004.docx]

**Table S1**. Means and standard deviations of body mass index by 5 year age band and sex used to generate standardised BMI in the CGPS and CCHS.

|  | **CGPS** | | **CCHS** | |
| --- | --- | --- | --- | --- |
|  | **Male** | **Female** | **Male** | **Female** |
| **5 year age band** | **Mean (SD)** | **Mean (SD)** | **Mean (SD)** | **Mean (SD)** |
| 20-24 | 24.0 (3.7) | 23.7 (4.7) |  |  |
| 25-29 | 25.5 (3.9) | 24.2 (4.4) | 23.5 (3.1) | 22.5 (3.1) |
| 30-34 | 26.0 (3.7) | 24.4 (4.4) | 23.8 (2.7) | 22.3 (2.8) |
| 35-39 | 26.1 (3.6) | 24.5 (4.7) | 24.0 (3.4) | 22.9 (3.0) |
| 40-44 | 26.4 (3.6) | 24.9 (4.5) | 23.8 (2.4) | 22.6 (3.2) |
| 45-49 | 26.8 (3.9) | 25.2 (4.6) | 24.4 (3.2) | 23.5 (3.0) |
| 50-54 | 26.8 (3.8) | 25.4 (4.4) | 24.4 (3.6) | 23.6 (4.1) |
| 55-59 | 27.2 (3.9) | 26.0 (4.9) | 24.6 (3.5) | 23.5 (3.9) |
| 60-64 | 27.4 (3.9) | 26.1 (4.7) | 25.1 (3.4) | 23.3 (3.8) |
| 65-69 | 27.3 (3.7) | 26.3 (4.7) | 25.5 (3.6) | 23.8 (4.1) |
| 70-74 | 27.3 (3.7) | 26.6 (4.5) | 25.4 (3.5) | 24.0 (4.0) |
| 75-79 | 26.8 (3.6) | 26.3 (4.4) | 25.4 (3.3) | 24.7 (4.3) |
| 80-84 | 26.5 (3.5) | 26.1 (4.3) | 26.2 (3.4) | 25.4 (5.0) |
| 85-99 | 26.0 (3.3) | 25.5 (4.2) | 25.9 (2.8) | 24.8 (3.6) |
| Overall | 26.9 (3.8) | 25.7 (4.6) | 25.2 (3.3) | 24.1 (4.0) |
