# Supplementary material for: The Effect of Elevated Body Mass Index on Ischemic Heart Disease Risk: Causal Estimates from a Mendelian Randomisation Approach
Source: PLoS Med. 2012 May 1;9(5):e1001212. doi: 10.1371/journal.pmed.1001212 (PMC3341326; doi:10.1371/journal.pmed.1001212)
Supplement: Table S2 — Associations of potential confounders with standardised BMI in the three studies. (DOCX) [file pmed.1001212.s005.docx]

**Table S2** . Associations of potential confounders with standardised BMI in the three studies.

|  | **CGPS** | **CCHS** | **CIHDS*** |
| --- | --- | --- | --- |
|  | Coefficient (95 CI) on linear regression | | |
| Sex | 0.002 (-0.01, 0.02) | 0.00 (-0.04, 0.04) | 0.02 (-0.03, 0.07) |
| Ever smoked | 0.02 (0.01, 0.04) | -0.17 (-0.20, -0.13) | 0.02 (-0.03, 0.07) |
| Drinking | -0.27 (-0.29, -0.25) | -0.10 (-0.14, -0.06) | -0.24 (-0.30, -0.18) |
| Age (yrs) | 0.0001 (-0.001, 0.001) | 0.004 (0.003, 0.006) | -0.001 (-0.003, 0.0004) |
| Education | -0.15 (-0.16, -0.14) | -0.19 (-0.22, -0.16) | -0.13 (-0.17, -0.09) |
| Income | -0.11 (-0.12, -0.10) | -0.03 (-0.06, -0.01) | -0.08 (-0.11, -0.05) |
| Event time | 0.005 (0.001, 0.009) | -0.024 (-0.028, -0.020) | -0.013 (-0.030, 0.004) |

*BMI, education, income, IHD event, only available for CIHDS controls.

Drinking represented by <14/21; >=14/21 units per week for women/men at the time of examination.

Education represented by years schooling, <10; >=10-<13; >=13.

Income represented by annual income, <100,000kr; 100,000-400,000kr; 400,000-600,000kr; >600,000kr.

Event time is absolute difference between age at IHD event and age at measurement of BMI (years)
