# Supplementary material for: The Effect of Elevated Body Mass Index on Ischemic Heart Disease Risk: Causal Estimates from a Mendelian Randomisation Approach
Source: PLoS Med. 2012 May 1;9(5):e1001212. doi: 10.1371/journal.pmed.1001212 (PMC3341326; doi:10.1371/journal.pmed.1001212)
Supplement: Table S3 — Associations of potential confounders with IHD in the three studies. (DOCX) [file pmed.1001212.s006.docx]

**Table S3**. Associations of potential confounders with IHD in the three studies.

|  | **CGPS** | **CCHS** | **CIHDS*** |
| --- | --- | --- | --- |
|  | Odds ratio (95 CI) on logistic regression | | |
| Sex | 1.98 (1.85, 2.12) | 1.55 (1.40, 1.71) | 3.15 (2.91, 3.42) |
| Ever smoked | 1.98 (1.84, 2.14) | 1.61 (1.45, 1.78) | 1.06 (0.98, 1.15) |
| Drinking | 1.03 (0.96, 1.11) | 0.98 (0.89, 1.08) | 0.19 (0.17, 0.21) |
| Age (yrs) | 1.07 (1.07, 1.07) | 1.06 (1.05, 1.06) | 1.05 (1.05, 1.05) |
| Education | 0.45 (0.43, 0.48) | 0.41 (0.38, 0.45) | NA |
| Income | 0.50 (0.47, 0.52) | 0.80 (0.74, 0.85) | NA |
| Event time | 1.73 (1.69, 1.76) | 0.79 (0.78, 0.79) | NA |

*Education, income, IHD event, only available for CIHDS controls (hence “NA”).

Drinking represented by <14/21; >=14/21 units per week for women/men at the time of examination.

Education represented by years schooling, <10; >=10-<13; >=13.

Income represented by annual income, <100,000kr; 100,000-400,000kr; 400,000-600,000kr; >600,000kr.

Event time is absolute difference between age at IHD event and age at measurement of BMI (years)
